# Interdisciplinary simulation scenario in nursing education: Humanized childbirth and birth*

**DOI:** 10.1590/1518-8345.3681.3286

**Published:** 2020-06-19

**Authors:** Luciana Mara Monti Fonseca, Juliana Cristina dos Santos Monteiro, Natália Del’Angelo Aredes, Juliana Villela Bueno, Aline Natália Domingues, Verónica Rita Dias Coutinho, Rui Carlos Negrão Baptista

**Affiliations:** 1 Universidade de São Paulo, Escola de Enfermagem de Ribeirão Preto, PAHO/WHO Collaborating Centre at the Nursing Research Development, Ribeirão Preto, SP, Brazil.; 2 Universidade Federal de Goiás, Faculdade de Enfermagem, Goiânia, GO, Brazil.; 3 Escola Superior de Enfermagem de Coimbra, Coimbra, Portugal.

**Keywords:** Simulation, Humanized Delivery, Obstetric Nursing, Neonatal Nursing, Education, Nursing, Teaching, Simulação, Parto Humanizado, Enfermagem Obstétrica, Enfermagem Neonatal, Educação em Enfermagem, Ensino, Simulación, Parto Humanizado, Enfermería Obstétrica, Enfermería Neonatal, Educación en Enfermeira, Enseñanza

## Abstract

**Objective:**

to develop and validate with a panel of *experts* a scenario of maternal-child clinical simulation, related to humanized childbirth and birth.

**Method:**

methodological study based on the Jeffries framework and standardized guides of the International Nursing Association for Clinical Simulation in Learning, which used analysis with descriptive statistics for general aspects of adherence to the aforementioned guide and inferential statistics for validating the *checklist* of actions through the Intraclass Correlation Coefficient (ICC).

**Results:**

the scenario contains learning objectives, necessary resources, *prebriefing* and *debriefing of guidelines, description of the simulated situation, participants and roles, and checklist of expected actions.* The validation obtained an agreement level above 80% in all aspects evaluated by 31 *experts*, highlighting realism of the environment and setting, vital sign parameters, alignment with scientific literature and encouragement of critical thinking and problem solving. In addition, the *checklist* of actions was validated with 0.899 agreement among *experts*, statistically analyzed by the ICC and Cronbach’s alpha 0.908 (95% confidence interval).

**Conclusion:**

the simulated scenario on humanized childbirth and birth can strengthen the articulation between women’s and children’s health disciplines, and was validated by *experts*.

## Abstract (pt)

**Objetivo:**

desenvolver e validar junto a um painel de *experts* um cenário de simulação clínica materno-infantil, relacionado ao parto e nascimento humanizados.

**Método:**

estudo metodológico baseado no referencial de Jeffries e guias padronizados da *International Nursing Association for Clinical Simulation in Learning*, que utilizou análise com estatística descritiva para aspectos gerais de adesão aos guias supracitados e inferencial para validação do *checklist* de ações por meio de *Intraclass Correlation Coeficient* (ICC).

**Resultados:**

o cenário contém objetivos de aprendizagem, recursos necessários, orientações de *prebriefing* e *debriefing*, descrição da situação simulada, participantes e papéis, e *checklist* de ações esperadas. A validação obteve nível de concordância superior a 80% em todos os aspectos avaliados por 31 *experts*, destacando realismo do ambiente e cenário, parâmetros de sinais vitais, alinhamento com a literatura científica e estímulo ao pensamento crítico e resolução de problemas. Ainda, foi validado o *checklist* de ações com concordância de 0,899 entre *experts*, analisado estatisticamente pelo teste de ICC e alpha de Cronbach 0,908 (intervalo de confiança 95%).

**Conclusão:**

o cenário simulado sobre parto e nascimento humanizados pode fortalecer a articulação entre as disciplinas de saúde da mulher e da criança, e foi validado por *expert*s.

## Abstract (es)

**Objetivo:**

desarrollar y validar con un panel de expertos un escenario de simulación clínica materno-infantil, relacionado con el parto y el nacimiento humanizados.

**Método:**

estudio metodológico basado en el marco de Jeffries y guías estandarizadas de la *International Nursing Association for Clinical Simulation in Learning*, que utilizó análisis con estadísticas descriptivas para los aspectos generales de la adhesión a las guías mencionadas e inferenciales para validar la *checklist* de acciones a través del *Intraclass Correlation Coeficient* (ICC).

**Resultados:**

el escenario contiene objetivos de aprendizaje, recursos necesarios, orientaciones de *prebriefing* y *debriefing*, directrices, descripción de la situación simulada, participantes y roles, y *checklist* de las acciones esperadas. La validación obtuvo un nivel de acuerdo superior al 80% en todos los aspectos evaluados por 31 expertos, destacando el realismo del entorno y el entorno, los parámetros de los signos vitales, la alineación con la literatura científica y el estímulo del pensamiento crítico y la resolución de problemas. Además, el *checklist* de acciones fue validado con un acuerdo de 0,899 entre *expertos*, analizada estadísticamente por la prueba ICC y el alfa de Cronbach 0,908 (intervalo de confianza del 95%).

**Conclusión:**

el escenario simulado sobre el nacimiento humanizado y el parto puede fortalecer la articulación entre las disciplinas de salud de mujeres y niños, y fue validado por *expertos*.

## Introduction

The complexity of the health situations that nursing professionals face in their daily lives requires a set of knowledge, skills and attitudes that must be mobilized in an articulated manner to address the health needs of the population^(^ 1 ^)^.

The National Curricular Guidelines for Nursing determine that learning is centered on the student as an active subject in this process, with the teacher having the role of facilitator and mediator^(^ 2 ^)^. This document directed curricular changes in the pedagogical projects of courses across the country, and its new preliminary version - approved by the National Health Commission in 2018 - shows elements that are compatible with this aspect, highlighting it even more.

However, for these guidelines to be translated into improvements in the teaching-learning process, it is necessary to invest in training based on active methodologies, which enable meaningful learning^(^ 3 ^)^.

In this context, clinical simulation stands out as a strategy aligned with the active pedagogy model, providing the student with the opportunity to develop complex learning by experiencing realistic situations in a safe and risk-free environment^(^ 3 ^)^. Through simulated practices, the student health professional can improve technical, communication and also behavioral skills, develop critical observation, learn to work in a team, exercise clinical reasoning and decision-making^(^ 1 ^)^.

Clinical or realistic simulation has an important educational attribute in improving the performance of students in practice settings, as it allows the execution of possible to make mistakes without harming themselves and others, reflecting about the error and define new strategies with a view to success before being subjected to care practice^(^ 4 ^-^ 5 ^)^.

Another important advantage of simulation, which in particular motivated the realization of this work, is the possibility of an interface between contents and themes that are generally fragmented in different disciplines. As it allows the articulation of contents in an interdisciplinary perspective, simulation is highlighted in competence education and in the exercise of clinical reasoning with a view to comprehensive health care. Thus, the objective of this research was to develop and validate a simulation scenario in the maternal and child area related to humanized birth and birth.

## Method

Methodological research for the development of a clinical simulation scenario in an interdisciplinary perspective and validation with *experts* on the topic of women’s and children’s health, held in the first quarter of 2019.

For the development of the simulation scenario, we adopted the framework proposed by Jeffries^(^ 6 ^)^ which consists of the following elements: Theme identification, simulation objectives, participants, scenario and *debriefing* process, in addition to the elaboration guides standardized by the International Nursing Association for Clinical Simulation in Learning (INACSL)^(^ 7 ^)^.

The theme “humanized childbirth” arose from the relevance of articulating the knowledge of care for women and children during childbirth and birth, problematizing real situations in a simulated laboratory environment. This scenario was developed as an activity of a training course for teachers, given at the institution where the research was carried out, by European simulation specialists. The scenario was initially presented to the participants of this course and, based on this activity, the scenario was improved with the suggestions of the participants and specialists teaching the course for teachers.

The scenario developed is liable to be conducted by teachers and facilitators from both areas, who generally separate themselves into disciplines in health care courses, but which are intrinsically related in the care process.

For the validation of this scenario, we offer an update course on the humanization of obstetric and neonatal care in which the participants were invited to validate the simulation scenario, with the information that the non-acceptance would not imply continuity of participation in the course, nor would it represent burden of any kind. All the guests accepted and signed the Free and Informed Consent Form. The study was approved by the Research Ethics Committee (CAAE: 02457118.9.0000.5393) official letter no. 3.134.086/2019, on February 6, 2019 and followed all ethical precepts provided for by Resolution 466/2012.

After acceptance, the course participants were divided into three groups (with 10, 10 and 11 participants) for the simulation, and in each group, two volunteers volunteered to perform the performance in the scenario, whose average duration was 10 minutes. As conventionally established in the simulation, the other members of the groups observed and participated in the *debriefing* stage.

Participating in the validation stage there were 31 nursing specialists in women’s and children’s health, being teachers, nurses working in health services with an emphasis on maternal and child care and graduate students in this same area of knowledge, a number considered excellent for the validation process^(^ 8 ^)^.

Validation took place in a Simulation Center and the developed scenario was of high conceptual, emotional and environmental fidelity, with experienced and specially trained actors and facilitators for the research scenario.

Thus, for the data collection of the present study, we developed an instrument from a robotic simulation validation work^(^ 9 ^)^, from the Bay Area Simulation Collaborative (BASC) group for validating scenarios^(^ 10 ^)^ and the Simulation Design Scale, translated and validated into Portuguese^(^ 11 ^)^, and we also include an open space for expert comments.

To improve the scenario and the instrument developed prior to the data collection, we conducted a pilot study among the facilitators, actors and invited graduate students, totaling six participants, at which time the actors’ rehearsal and alignment between the facilitators was carried out, as well as management of the high fidelity obstetric simulator used in the simulation.

After completing the simulation (*prebriefing,* scene and *debriefing*), the participants completed the validation instruments.

For the validity, the agreement percentage was used, which considered the minimum of 80% satisfactory among the *experts* ^(^ 8 ^)^. In order to guarantee the validation of the instrument, an agreement analysis was conducted using SPSS version 21.0 in order to measure the consistency of the evaluators’ decisions^(^ 12 ^)^ through the Intraclass correlation coefficient - ICC test. ICC is a statistical method that allows measuring reliability of evaluations reflecting both the degree of correlation and agreement among evaluators^(^ 13 ^)^ and, to meet the purpose proposed in the study, the absolute agreement analysis was used, in order to measure whether the different experts would attribute similar scores using the proposed instrument.

The cut-off points of the ICC analysis generally vary depending on the reference, but always with values between 0 and 1, indicating a high correlation the closer to 1 and a low correlation the closer to 0. In this study, we adopted the framework that defines: ICC <0.4 as weak; 0.59> ICC> 0.4 as regular; 0.74>ICC>0.59 as good and 1.0> ICC> 0.74 as excellent^(^ 14 ^)^.

In addition, the descriptive analysis evaluated the simulation scenario regarding the contained information that guides the student in solving the problem situation, alignment with scientific evidence, realism, resources used, level of difficulty and *debriefing*.

## Results

As a result of the first objective of this study, that of developing the simulation scenario, it was called “Humanized childbirth and birth” and had the following learning objectives: Offering humanized assistance during childbirth and birth and clinically evaluate women and newborns (NB), in order to encourage skin-to-skin contact and breastfeeding in the first hour of life.

The simulated scene presents, in general, a primigravid, adolescent parturient, accompanied by her sister in the delivery room during the period of childbirth. The parturient is in a gynecological position, being attended by a health professional specializing in obstetrics with disrespectful conduct and out of alignment with good humanization practices in care. The newborn is born flushed, crying and sneezing, Apgar 10/10 and is received by the health professional represented by an actor who recommends placing the baby in the warm crib and calling the pediatrician. At this point, the simulation has two predicted outcome points: 1- The volunteer participant on the scene suggests that due to the good conditions, the newborn is placed next to the mother for skin-to-skin contact and breastfeeding, or 2- The volunteer participant on the scene places the newborn in the heated crib and calls the pediatrician.

As for the resources needed to implement the simulation, we recommend what was developed and validated in this study, which consists of: High-fidelity obstetric simulator with NB simulator, an actor or actress to represent the health professional who conducts the delivery, an actor or actress to represent the pediatrician who can be called on to the scene and an actress to represent the parturient’s sister. The decision to change the companion is free and does not interfere with the outcome of the scenario, and may be the person the parturient wishes, such as her spouse, mother, sister or brother, friend or friend, in adherence to Law No. 11.108, of April 7 2005^(^ 15 ^)^.

We highlight the importance of the obstetric simulator allowing the perception of the participants that a normal birth is in fact occurring, made possible by the baby’s exit through lubrication of the simulated birth canal. The lubricant applied in the simulator must be compatible with the maintenance of the material of the simulator, avoiding risks of depreciation of the mannequin.

The figure of the pediatric specialist medical professional was not originally included in the scenario, but after validation (described below), the experts suggested their inclusion to strengthen the resemblance to the real birth centers.

It is necessary to have a heated crib in the simulated environment, an auxiliary table with materials such as fluids for intravenous infusion and venous access materials, glucometer with tapes, oximeter, stethoscope, sphygmomanometer, sterile fields and water filter with cups. Still in the perspective of environmental support, it is worth noting the importance of a physical medical record with data on the parturient, partogram, a form with the variables of the Apgar evaluation and for recording the delivery data, which must be prepared by the teachers in advance.

To perform the simulation, the first moment was the prebriefing, which includes prior preparation for what will be experienced in the scenario based on the theme. Thus, the participants previously received scientific references for the study of childbirth and birth with a focus on the humanization of care. At the time of the simulated activity with the participants, at the Simulation Center, the prebriefing followed the following steps: Agreement of the rules, the roles to be played, mutual respect and confidentiality. Thus, in the prebriefing, in addition to these items, we identified the experiences of the participants in previous simulations and in childbirth and birth, we clarified that the general objective of the scenario would be assistance in this context, we inform that the estimated scene time would be ten minutes and we present the environment and the functioning of equipment and mannequins. A moment was offered for the participants to familiarize themselves with the scenario. The prebriefing lasted five minutes.

Before the volunteer participant on the scene acted properly in the problematic situation, he participated in the shift by a colleague from the health unit who presented the data contained in Figure 1.

**Figure 1:** Data referring to the shift in the simulated humanized childbirth and birth scenario. Ribeirão Preto, São Paulo, Brazil

SHIFTING PASS
Simone, a 15-year-old primigravid parturient, with 8 cm dilation, is accompanied by her sister in the delivery room. She mentions that she is experiencing severe pain during the contractions, but is confident about the vaginal delivery route decided in the prenatal period, which by the way followed all the steps and recommendations of the Ministry of Health. BP = 128x80 mmHg, HR = 98 bpm, oxygen saturation = 97%. Without analgesia, without serum.

Immediately after the *prebriefing*, in the simulated scene, the role of the actors was established as follows:

- The companion should remain beside the simulated parturient holding her hand and speaking supporting words. If, after birth, the volunteer participant on the scene referred the baby to the heated crib as recommended by the doctor who conducted the delivery, the companion should ask if it is not possible to place the baby on the mother’s lap, as this was how she was informed during prenatal care;
- Parturient (simulator) having her voice represented by a facilitator should ask the volunteer participant on the scene during the expulsive period if she could drink water, claiming to be very thirsty; and if he could hold your hand, complaining of pain and asking professionals not to touch you anymore;
- Obstetrician professional would represent inappropriate conduct in the perspective of humanization, and underestimate the complaint of pain and thirst, responding that if he needs to know the dilation he will repeat the touch exam and, upon birth, guide the volunteer participant on the scene to place the newborn in the heated crib and calling the pediatrician, without putting him in contact with the mother;
- Pediatric medical professional would be further removed from the main focus of the scenario, and may even be in another room to be triggered by those involved in the scenario, only in view of the outcome 2 that involves calling the pediatrician before putting the binomial in contact.

For the development of the simulated scenario, the checklist for minimum expected actions, which was validated by the experts, is shown in Figure 2 and brings the possibilities of responses by the evaluators considering, in addition to the performance or not, whether it was correct or incorrect and whether it was performed at the appropriate time or late by the volunteer on the scene.

**Figure 2:** Checklist of expected actions of volunteer participants on the scene during the simulated humanized childbirth and birth scenario. Ribeirão Preto, SP, 2019

Checklist for expected actions	1*	2^†^	3^‡^	4^§^
Perform data collection in the medical record (delivery plan)				
Introduce yourself to the parturient and companion				
Meet the parturient's needs				
Identify NB signs that indicate Apgar score				
Present autonomy and security to receive the newborn without the need to call the pediatrician				
Put the newborn in immediate skin-to-skin contact with the woman and encourage the initiation of breastfeeding				
Dry the newborn with a sterile field				
Cover the NB with a dry field				
Continue to encourage breastfeeding				

* 1 = Did not perform;

† 2 = Incorrectly performed;

‡ 3 = Performed in a correct way at the incorrect time;

§ 4 = Performed correctly at the correct time

After the simulated scene, the *debriefing* was conducted focusing on the discussions related to humanized care to the parturient and her family, to nursing care in childbirth at usual risk, to the evaluation of the newborn and Apgar score, the need or not for interventions depending on the condition of the binomial, stimulation of skin contact the skin between mother and baby as soon as clinically possible, and preferably immediately after birth and breastfeeding in the first hour of life.

In response to the second objective of the study, the scenario validation with experts included 31 nurses, mostly aged between 21 and 40 years old (93.5%) and a complete postgraduate course (77.4%), especially specialization ( 41.9%), followed by master’s (29%) and doctorate (6.4%). The significant majority of nurses who participated in this study had more than four years since graduation (n = 26; 83.8%), with the specialty area well divided between obstetric nursing and women’s health (n=13; 41.9%) and neonatology, maternal-infant and child health (n=10; 32.25%), with specialists also participating in intensive care and family health.

At the time of data collection, the main area of expertise was health care (n = 23; 74.1%), and some of them were also enrolled in graduate courses. Two professors with doctorate degrees took part in the scenario validation process.

The findings revealed that the experts considered the simulation adequate unanimously in terms of aspects: Realism, support offered to participants during the course of the activity as foreseen in the written scenario, learning objectives compatible with the simulated situation and the type of simulator used in the laboratory.

Among the 20 criteria evaluated, only two achieved an adequacy of less than 90%, with the summary of the case present in the scenario assessed as adequate by 25 experts (80.6%) and partially adequate by the others; and the data provided to the participant during the simulation assessed as adequate by 27 experts (87.1%) and as partially adequate by the others.

Only one aspect was considered inadequate by one of the nurses regarding the item that evaluated the opinion of alignment with the available scientific evidence, but, upon analysis of his free comment in the questionnaire, we found that the inconsistency was not in what the scenario advocates as expected action, but in the way the simulation actress acted. Thus, we excluded this expert in the analysis of this item, as it was expected that the simulation actress would problematize deviations from good clinical practices, precisely so that the participant could identify inconsistency with the scientific literature.

**Figure 3:**
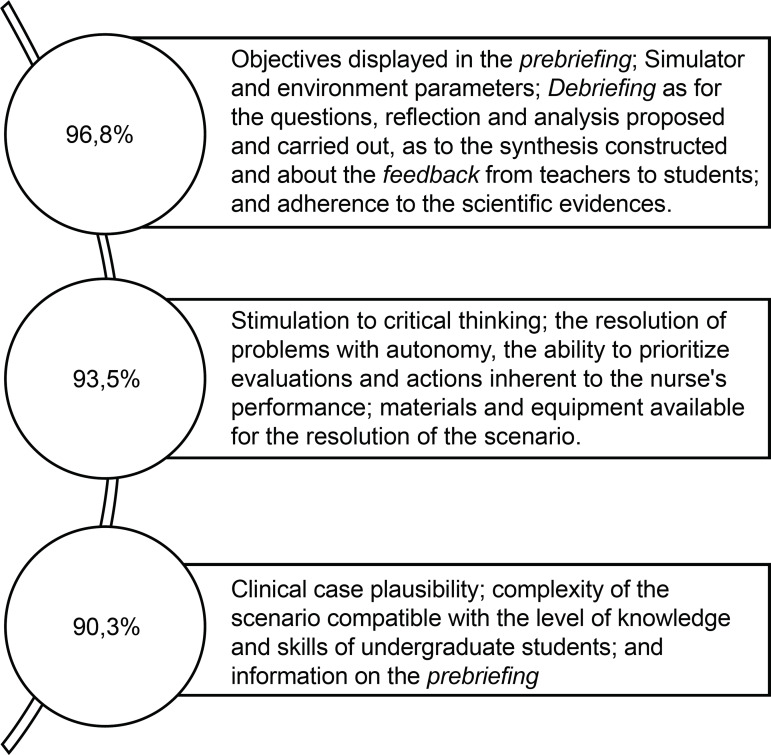
Items evaluated by experts and frequency of full approval by experts between 90.3% and 96.5%. Ribeirão Preto, SP, Brasil, 2019

Figure 3 shows other aspects evaluated and the level of adequacy conferred by specialist nurses with a frequency of approval between 90.3% and 96.5%.

Regarding the evaluation of the comments recorded by the participant experts, we display below the main content of the speeches, which were classified in the categories 1- Strengths of the scenario and 2- Suggestions for improvement.

The positive aspects most highlighted by the experts were the ability of the scenario to bring “sufficient elements for critical assessment and decision making”, and the classification of the simulation as being “realistic and intense”, reflecting real challenges of the nurse’s daily life in the delivery room, such as the rupture the hierarchy of powers of health professionals with medical training over others and the difficulty of strengthening the parturient’s role in childbirths conducted by professionals who do not adopt humanized conduct.

With regard to suggestions for improvement, the experts reinforced the importance of using the medical record to spend the shift on the scene and include more information on the parturient, such as food and hydration, for example.

Still, they suggested a reduction in the period of expulsive delivery, starting the scene before the period of expelling delivery to allow the professional to introduce herself to the parturient and companion, not to hold the field early before a clear sign that the baby is in the vaginal canal and include the pediatrician in the scene.

We emphasize that other suggestions were recorded and are classified as improvement not of the proposed scenario exclusively, but of the recommended conduct in humanized delivery situations such as: Informing on the shift change the parturient’s desire regarding positioning during delivery, decreased brightness and noise in the environment, late clamping of the umbilical cord, offering more time for breastfeeding in the first hour of life.

In this context, the scenario was improved by adhering to the suggestions in the following elements:

1- Inclusion in the medical record of more information about the parturient, 2- Reduction of the period of expulsive delivery; 3- The actor or actress who conducts the delivery will present the sterile field only when the baby crowns; and 4- Inclusion, in the description of the scenario, of the recommendation to mention that there is a pediatrician available to be called in that delivery room upon demand, or else present since the beginning of the scenario near the heated cradle (but without the function provided for in it, given the presupposed only to confer greater realism).

The other suggested elements were not considered in the adaptation of the scenario because they consisted of or in the action of the simulation participant and it varies depending on his clinical conduct; or because they wanted precisely to intervene in adversity to promote better conditions for the parturient and companion (such as the control of light and noise in the delivery room).

Regarding the level of agreement among the experts verified through the ICC we found an excellent agreement index (ICC = 0.899) and alpha of Cronbach = 0.908.

This finding indicates that the 31 evaluators had a high correlation of response in the evaluated items, when analyzing the simulated scenario, which corresponds to an agreement on the perception of the performance or not of the activities by those involved during the action.

Through this analysis it was possible to validate the checklist of expected actions by the participants during the scenario.

## Discussion

Nursing courses linked to Higher Education Institutions (HEIs) in Brazil have been challenged to incorporate interdisciplinary theoretical and practical activities in their curricula, especially since the publication of the National Curriculum Guidelines (NCG)^(^ 2 ^)^, in order to overcome the fragmentation inherited from the traditional school.

Through interdisciplinarity, which consists of the articulation of knowledge from different disciplines or subjects with a focus on developing students’ abilities to question and intervene in the world^(^ 16 ^)^, it is possible to expand the learners’ view beyond what each list of content offers, leading them to better understand how problems are identified and how knowledge is articulated to solve them.

The simulation scenario developed and validated aims to contribute to the interface between disciplines of the nursing course, and others in the health area that work in childbirth and birth, articulating the areas of obstetrics and neonatology, or women’s health and children’s health, in a more holistic perspective of the phases of life and phenomena of human reproduction.

Initiatives like this have been encouraged by documents regulating nursing courses in the country^(^ 1 ^)^ and simulation, in general, draws attention as a strategy to strengthen integrated, critical and resolutive learning^(^ 17 ^-^ 18 ^)^.

In addition to the articulation of women’s health and children’s health content in nursing, the scenario has potential for interprofessional use, expanding the concept of interdisciplinarity to other professional categories in the health field that work with the common goal of promoting safe and comprehensive care to the patients and their families^(^ 19 ^)^.

Inter-professional teaching in courses promoted by HEIs is encouraged by the World Health Organization itself, so that it makes possible the realization of a collaborative practice and with an important advance in communication^(^ 20 ^)^.

Although admittedly necessary, both interdisciplinarity and interprofessionality, it is still challenging to break the barriers of fragmented teaching in HEIs.

On the other hand, advances have been made with the reformulation of pedagogical projects for courses and from the commitment of students and teachers in the use of active methods and strategies, such as the mapping of competences^(^ 21 ^)^, the curricula integrated with activities of interface of the basic area with the specific area^(^ 22 ^)^ and curricula that incorporate the simulation^(^ 18 ^)^.

The results of the validation were quite positive and the experts’ suggestions added greater quality to the scenario, strengthening its realism and providing more specific information related to humanized childbirth - a topic that is very important in the current context of the country, in which it seeks to improve the quality of care to women throughout the pregnancy-puerperal cycle, in order to reduce the maternal mortality rate, which is currently 60 deaths per 100,000 live births^(^ 23 ^)^.

Therefore, it is necessary to advance in aspects of universal access to quality health services that are effective and safe, and that offer reliable guidance to women, favoring their autonomy for informed decision-making during care during this period, so that they have their rights respected by health professionals^(^ 24 ^)^.

Still in the national epidemiological context, infant mortality was reduced during the analysis of the millennium goals, reaching around 15 deaths per 1000 live births in 2015^(^ 25 ^)^.

Despite national and global advances, it is important to note that 45.1% of infant deaths occur in the neonatal period worldwide^(^ 26 ^)^.

Of these deaths, the second leading cause of neonatal mortality, that is, from the day of birth to 28 days of life, is related to intrapartum events (10.7% of deaths in children under five years old), highlighting the need to strengthen teaching in this area.

The integration of teachers from different areas in a simulated environment has the potential to favor the understanding of students and health professionals about the end to end integral care binomial.

Combining the contents of nursing care for low-risk normal childbirth and the reception and clinical evaluation of the newborn, the first golden hour and humanization, the scenario developed and validated potentially breaks with fragmentation and promotes curricular integration.

We emphasize the importance of the stage of validation and dissemination of the tested scenario, including the instrument to be used by facilitators in conducting this scenario in their HEIs, considering that the knowledge of methods, models and standardized simulation guides by teachers are fundamental aspects for its implementation in the curriculum^(^ 27 ^)^.

Studies reporting the use of simulation in the area of women’s and children’s health suggest important achievements with students, such as increased perception of self-confidence to conduct births^(^ 28 ^)^, teamwork and patient-centered care during care for women during parturition^(^ 29 ^)^ and better knowledge about NB clinical evaluation^(^ 30 ^).^

Pondering the benefits of implementing simulation as a learning strategy from the perspective of active methods, we emphasize that the availability of high technology is not necessary to guarantee the success of the activity.

Recent research carried out in Guatemala analyzed the impact of a simulation course with few technological resources conducted in situ and obtained results that encourage the use of simulation in this context^(^ 29 ^)^.

Another study developed in situ with teachers of kindergarten and elementary school I showed that the simulation contributed to increase the self-confidence of these participants to manage health problems in schools, reinforcing the relevance of using this strategy even outside simulation centers^(^ 31 ^)^.

Thus, for HEIs that do not have a high fidelity environment, conducting the simulation in health units - when available for this purpose - can be an interesting proposal.

Or, even in a laboratory with little equipment or without a high-fidelity simulator, it is possible to implement the strategy with a portable simulator that has significantly lower cost and also presents favorable results in the teaching-learning process^(^ 28 ^)^.

Even from the simulation that mixes simulated anatomical parts and an actor or actress representing the patient, it is possible to strengthen the aspect of interpersonal communication^(^ 28 ^)^, impaired in the non-verbal aspect when the student must communicate with a mannequin^(^ 32 ^)^.

Clinical simulation allows the student to experience the stresses of participating in the health team during childbirth and being responsible for assessing health needs and acting quickly, efficiently and in accordance with what is recommended.

The experts who took part in this study, suggested to include in the list of expected actions the late clamping of the cord, however this was not incorporated due to the validation process being underway and because the clamping, in a realistic way, would be under the responsibility of the professional interpreted by an actor and not by the student on the scene.

In the prebriefing, which displays guidelines and information on the theme through videos, texts and other sources immediately before the simulated scene, participants should be guided to the next steps of the activity.

The prebriefing displays a review of the objectives of the scenario, guides on the use of equipment, mannequins, the roles to be developed by each person in the scene, the time of execution of the scenario and the situation to be experienced^(^ 7 ^,^ 33 ^)^, recommendations that were followed in this study.

The purpose of the prebriefing is to establish a psychologically safe environment for the simulation participants, with the establishment of a work “contract” for the activities^(^ 7 ^)^.

After the prebriefing and the simulated scene, the debriefing took place, an important moment of reflection and discussion to consolidate learning, which contributes to improving the performance of students apprentices in the real practice of health care^(^ 34 ^)^.

The debriefing was structured according to the emotional, behavioral and cognitive stages and was mediated by the facilitator, who initially sought the verbal expression of the participants who were on the scene and, subsequently, the expression of the other members of the group^(^ 35 ^)^.

For operationalization purposes, we can divide the debriefing in the following phases:

Phase 1 - Meeting: Conducted in order to listen to the participant, using the following initial question: How did you feel taking care of the binomial? (planned time: 5 minutes);
Phase 2 - Analysis: This phase seeks to facilitate reflection and analysis of the participants’ actions, based on the following questions: What positive actions did you take? What would you do differently if you had another opportunity? What can you take as an apprenticeship? (planned time:15 minutes);
Phase 3 - Summary: The objective is to identify and analyze the situations apprehended in the light of the scientific evidences (estimated time:5 minutes).

The actions recommended for the care of the binomial in the situation of childbirth and birth were addressed in the debriefing, as suggested in the section Results of this work.

We emphasize that the debriefing is necessary and can be carried out in different ways, from different references, but we suggest the format we implemented in this study, considering the positive evaluation by the participants.

Although a duration forecast is provided for each phase^(^ 35 ^)^, the time used for the debriefing depends on the situations of the proposed simulation scenarios, the objectives of the simulation, the facilitator and the students. In the present scenario, the debriefing was 20 minutes long.

The limitations of the study refer to the population participating in the validation, which although it is aligned with what is recommended in the literature (experts in the area), we felt the need to use this scenario with undergraduate students, target audience of the simulation, to have their opinion and suggestions about the elements of each of the stages of the strategy, as well as about the reliability, mediation, learning and motivation.

The contribution of the study refers to the possibility of improving significant learning in the management of humanized birth and birth together with the training of students and health/nursing professionals.

This investigation contributes to the scientific advancement of research on nursing simulation, through the methodological rigor adopted.

This is because the study presents not only the theme of a case that does not involve urgency and emergency (most contingent of simulated scenarios created in nursing), but a new look at a situation that often happens, unfortunately, in hospitals and maternity hospitals and that nurses’ decision-making is required to change reality.

In addition, the study presents the steps for the development and validation of interdisciplinary scenarios, strongly recommended by the World Health Organization.

## Conclusion

Simulation is a teaching-learning strategy with a strong potential for interdisciplinarity and benefits recognized in the scientific literature about the development of skills in the various branches of knowledge.

From the need to foster the articulation between knowledge and reduce curricular fragmentation, the scenario developed involves the areas of women’s and children’s health, in themes generally contained in the plans of these disciplines for nursing.

By proposing active student participation in assisting the binomial in humanized childbirth and birth, the scenario developed in the articulation of teachers in both areas and validated by 31 specialist nurses contemplates the theme of humanized childbirth, clinical assessment of the NB in the delivery room, promotion of breastfeeding in the first hour of life and skin-to-skin contact between mother and baby and can be successfully reproduced during students’ learning about the theme.
